# Prediabetes Induced by a Single Autoimmune B Cell Clone

**DOI:** 10.3389/fimmu.2020.01073

**Published:** 2020-06-18

**Authors:** Nathaniel Phillips, Eugene Ke, Amy Nham, Maximilian Seidl, Brent Freeman, Justin R. Abadejos, Changchun Xiao, David Nemazee, Manching Ku, Oktay Kirak

**Affiliations:** ^1^Department of Immunology and Microbial Science, The Scripps Research Institute, La Jolla, CA, United States; ^2^Salk Institute for Biological Studies, La Jolla, CA, United States; ^3^Institute of Pathology, Heinrich-Heine University and University Hospital of Düsseldorf, Düsseldorf, Germany; ^4^Division of Pediatric Hematology and Oncology, Department of Pediatrics and Adolescent Medicine, Faculty of Medicine, Medical Center - University of Freiburg, Freiburg, Germany

**Keywords:** diabetes, autoimmunity, B cell, animal model, pancreas, cloning, somatic cell nuclear transfer (SCNT), nucleic acid sensing

## Abstract

While B cells play a significant role in the onset of type-1 diabetes (T1D), little is know about their role in those early stages. Thus, to gain new insights into the role of B cells in T1D, we converted a physiological early pancreas-infiltrating B cell into a novel BCR mouse model using Somatic Cell Nuclear Transfer (SCNT). Strikingly, SCNT-derived B1411 model displayed neither developmental block nor anergy. Instead, B1411 underwent spontaneous germinal center reactions. Without T cell help, B1411-Rag1^−/−^ was capable of forming peri-/intra-pancreatic lymph nodes, and undergoing class-switching. RNA-Seq analysis identified 93 differentially expressed genes in B1411 compared to WT B cells, including *Irf7, Usp18*, and *Mda5* that had been linked to a potential viral etiology of T1D. We also found various members of the oligoadenylate synthase (OAS) family to be enriched in B1411, such as *Oas1*, which had recently also been linked to T1D. Strikingly, when challenged with glucose B1411-Rag1^−/−^ mice displayed impaired glucose tolerance.

## Introduction

Autoimmune Type-1 diabetes mellitus (T1D) is caused by the destruction of pancreatic insulin-secreting β-cells (1). Over 50 genes have been identified as risk factors in T1D (2, 3). However, the annual rise of T1D incidence seems to be impacted by the country of residence and additional non-genetic factors (4–6). Based on clinical and pathological studies, one of the main environmental triggers for the development of T1D has been identified as enteroviruses, such as Coxsackie Virus Type B (7–11).

The autoimmune attack on pancreatic β-cells is executed by both innate and adaptive immune cells (12). Among the adaptive immune cells, a major focus had been on CD4^+^ and CD8^+^ T cells. This is probably due to reports in the non-obese diabetic (NOD) mouse model showing that disease can be induced in recipient mice only by adoptive transfer of CD4^+^ and CD8^+^ T cells isolated from diabetic mice (13–16). In addition, T1D could be reversed in NOD mice by injection of a tolerogenic anti-CD3 antibody (17, 18). However, the contribution of B cells in T1D needs further exploration. While depletion of B cells by antibody injection or gene targeting lessened the T1D manifestation in NOD mice, B cells alone were not capable of inducing T1D upon adoptive transfer into recipient mice (19, 20). Clinical studies further support an essential role of B cells in T1D based on the observation that B cell-depleting treatments lead to an improvement in pancreatic β-cell function (21).

BCR transgenic mice represent an indispensable resource to improve our understanding of B cell functions in diseases. Various BCR transgenic mouse models had been generated to model the development and function of B cells on NOD background [for a detailed review see (22)]. For example, Ig3-83 recognizing H-2K^k^ and H-2K^b^ was used to demonstrate functional receptor editing in NOD (23). B cell anergy in NOD was studied using IgHEL and 125Tg BCR models recognizing hen egg lysozyme and human insulin, respectively (24, 25). Recently, a transgenic BCR model was generated recognizing the neuronal antigen peripherin, which contributed to T1D (26).

We had successfully created novel T_reg_ mouse models on pure NOD background by epigenetic reprogramming through somatic cell nuclear transfer (SCNT) (27, 28). Strikingly, these T_reg_ models provided new insights into the existence of distinct thymic T_reg_ subsets. Consequently, we reasoned that a novel SCNT-derived BCR mouse model from a physiological early pancreas-infiltrating B cell during the natural course of an autoimmune attack might uncover new mechanisms into the involvement of B cells in T1D.

## Materials and Methods

### Mice and Somatic Cell Nuclear Transfer

We generated B6D2F1 female mice as oocyte donors by crossing C57BL/6 with DBA/2 mice. Donor B cells were isolated from 6-week-old male NOD-Rag1^+/−^ for SCNT. SCNT procedure was performed as previously described (29, 30). B6D2F1 female mice were super-ovulated and their oocytes were isolated and enucleated. One donor B cell was transferred into one enucleated oocytes resulting in a total of 143 reconstructed SCNT embryos, which gave rise to two SCNT Blastocysts. One ES cell line was successfully derived and utilized to generate chimeric mice. NOD-B1411-Rag1^−/−^ mice were obtained through breeding with NOD-Rag1^−/−^ mice, and various genotypes were used for analysis as indicated. The Institutional Animal Care and Use Committee at the Scripps Research Institute had approved all animal experiments illustrated in this paper.

### BCR Identification

The BCR of B1411 was identified as previously reported (29). RNA was isolated from splenocytes of B1411-Rag1^−/−^ mice (Macherey Nagel). 5′-RACE was performed, and results were analyzed using the Ensembl Genome Browser (www.ensembl.org) and the international ImMunoGeneTics information system (www.imgt.org).

### Flow Cytometric Analysis and Cell Sorting

Single-cell suspensions of bone marrow, spleen, or peritoneal fluid were prepared and red blood cells were lysed. All analyzed mice were 6–12 weeks of age, unless indicated differently. Cells were incubated with various combinations of the following antibodies: CD3, CD4, CD5, CD8, CD19, CD21, CD23, CD24, CD62L, B220, AA4.1, CXCR5, FAS, GL7, Gr-1, IgD, IgM, PD1, and TCR-β (eBioscience and Tonbo). Samples were collected using FACS LSRII (BD) or sorted using FACSAria (BD), and data were analyzed with FlowJo software (Tree Star). Differences between groups were analyzed using unpaired *t*-test analysis (GraphPad Prism software).

### ELISA

Serum from WT NOD, B1411-Igμ^hom^Igκ^hom^ and B1411-Rag1^−/−^ mice were analyzed using the SBA Clonotyping System-HRP Kit according to manufacturer's protocol (SouthernBiotech). Briefly, the capture antibody was diluted 1:500 and coated on 96-well plates overnight. Serum of indicated mice was then incubated for 1 h at room temperature. After incubation with HRP-conjugated detection antibodies, TMB substrate was added to evoke the colorimetric response and sulfuric acid was used to stop the reaction. The optical density was read at a wavelength of 450 nm.

### Ca-Signaling

1 x 10^6^ cells of both WT NOD and B1411-Rag1^−/−^ splenocytes were prepared. Initially, WT or B1411 cells were stained with CFSE. Both populations were then mixed and stained with calcium indicator dye Indo-1 and kept in the dark on ice (Life Technologies). The samples were warmed in a 37°C water bath for 15 min prior to flow cytometric analysis. Baseline was determined for about 1 min, before the cells were stimulated with anti-Igμ or anti-Igκ. Ca-flux was recorded for ~8 min. Finally, Ionomycin was spiked into the cell suspension as positive control.

### *In-vitro* Nur-77 Assay

B Cells were isolated by MACS from spleen of WT NOD and B1411-Rag1^−/−^ mice and stimulated with anti-IgM. Cells were intracellularly stained by Nur77-PE 3–4 h after activation (eBioscience). Endogenous Nur-77 levels were determined using flow cytometry.

### Glucose Tolerance Test

WT NOD, B1411, and B1411-Rag1^−/−^ mice were initially fasted for 5 h. All mice were 10–14 weeks old at the time of experiment. Subsequently, mice were weighed and fasting glucose levels were determined. WT NOD, and B1411 mice that had already overt diabetes as judged by elevated fasting glucose levels (above 150 mg/dl) were excluded from this experiment. Mice were then injected i.p. with glucose (2 g glucose/kg body weight), and blood glucose measurements were then measured every 30 min after injection for a total of 2 h.

### mRNA-Seq and Data Analysis

We isolated total RNA by Macherey-Nagel Nucleospin RNA XS kit, and then RNA was processed into mRNA-Seq libraries using Illumina Truseq Stranded mRNA-Seq sample prep kit. First, total RNA was mixed with oligo-dT magnetic beads to select for mRNA. Then enriched mRNA was fragmented and reverse-transcribed. Subsequent cDNA was end-repaired, adenylated, adapter-ligated and PCR amplified. mRNA-Seq libraries were sequenced on Illumina HiSeq 2500 at single-end 50-bp (base pair), resulting 25–30 million reads per library. Sequencing reads were mapped to the mouse genome (mm 10, MGSCv38) using STAR (v2.2.0c) (31). The RNA-Seq data have been deposited in NCBI's Gene Expression Omnibus (GEO) and are accessible through GEO Series GSE114831 (will be publicly available upon publication). Gene expression was quantified using aligned reads to exons of RefSeq transcripts using HOMER (32) and differential gene expression was determined with edgeR (33) and plotted in Volcano Plot. Differentially expressing genes were analyzed by StringDB (34) to determine potential specific protein-protein interaction network.

### ATAC-Seq and Data Analysis

ATAC-Seq was performed as described previously (35) with modified nuclei isolation (36). Briefly, indicated cell populations were isolated by flow cytometric cell sorting. Nuclei were isolated using cell lysis buffer (10 mM Tris, 50 mM KCl, 60 mM NaCl, 5 mM MgCl_2_, 250 mM Sucrose, 0.5% Triton X-100, protease inhibitors). Isolated nuclei were resuspended in wash buffer (10 mM Tris, 50 mM KCl, 60 mM NaCl, 5 mM MgCl_2_, 250 mM Sucrose, protease inhibitors), layered on top of a sucrose cushion (30% sucrose v/v in Wash buffer) and centrifuged at 4,000 rpm for 20 min. Supernatant was discarded and pelleted nuclei were resuspended with transposition reaction buffer. Transposition reaction was carried out at 37°C for 30 min, then cleaned up by Zymo DNA columns and followed by PCR amplification using NEB Q5 mastermixes and Illumina Nextera indexed primers. ATAC-Seq data was aligned to the mouse genome (mm 10, MGSCv38) using bowtie2. Differential ATAC-Seq peak enrichment and Motif analyses were performed using HOMER. The ATAC-Seq data have been deposited in NCBI's Gene Expression Omnibus (GEO) and are accessible through GEO Series GSE114831 (will be publicly available upon publication).

## Results

### Generation of a Novel SCNT-Derived B Cell Model

**Figure 1:**
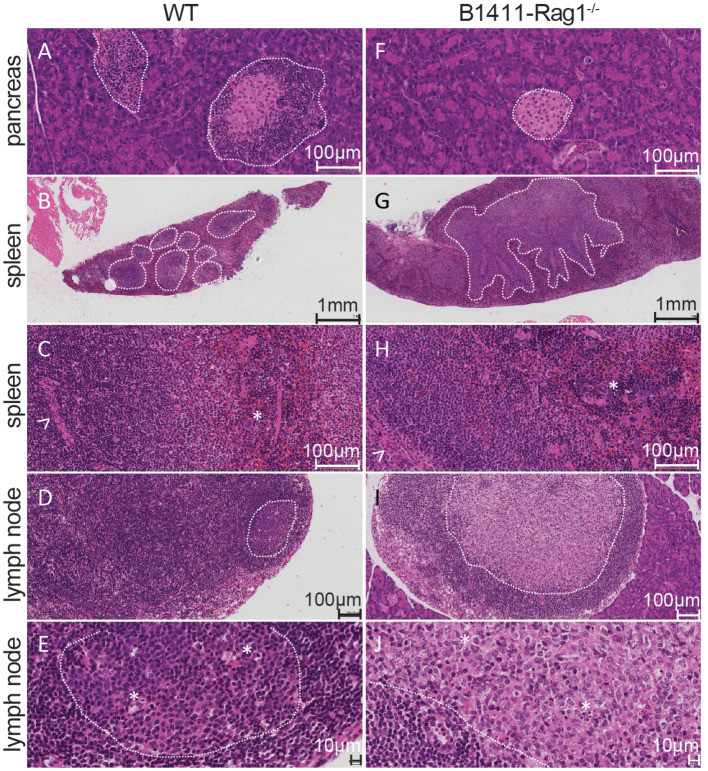
Histological comparison of SCNT-derived B1411 and WT NOD. **(A)** Severe insulitis with inflammatory lymphocytic infiltrations of islets (highlighted by dotted line). **(F)** Pancreas of B1411-Rag1^−/−^ with bland islet (dotted line). Spleen specimen of wildtype **(B,C)** and B1411-Rag1^−/−^ **(G,H)** mouse. The dotted lines highlight the white pulp, with the marginal zone in their outer areas, respectively. Normal, circular white pulp in the wildtype **(B)** compared to ill-defined, hyperplastic white pulp in B1411-Rag1^−/−^ **(G)**. **(C,H)** Show higher magnifications of spleen, displaying a normal composition at the cellular level, from the arteriolar (highlighted by arrowhead) sheath, ending with the red pulp with its hematopoietic cells (megacaryocyte highlighted by asterisk, respectively). **(D,E)** Lymph node with orderly maintained compartments such as germinal centers (dotted line) in WT NOD. **(I,J)** Absent germinal centers in lymph nodes of B1411-Rag1^−/−^. However, B1411-Rag1^−/−^ possessed large zones with brighter staining (dotted line). At higher magnifications, these zones in B1411-Rag1^−/−^ **(J)** show similarities with lymphocytes and tingible body macrophages (asterisks). The lymphocytic density is much higher in WT germinal center **(E)** compared to the bright, mixed cellular zone of B1411-Rag1^−/−^ **(J)**, which displays a much higher density of histiocytoid cells.

We had previously generated two novel SCNT-derived thymic T_reg_ model using donor cells from pure NOD background (27, 28). For an unbiased approach, we utilized NOD-Rag1^+/−^ mice in which the BCR IgH and IgL-locus were initially in wildtype configuration. In order to distinguish intravascular B cells from intra-pancreatic B cells, we intravenously injected a biotinylated CD45.1 antibody into the tail vein ~4 min prior to the isolation of the pancreas. Pancreas and infiltrating immune cells were then harvested using Collagenase P (37). B cells were then sorted from the pancreas of a 6-week-old male NOD-Rag1^+/−^ mice using flow cytometry and used as donor cells for SCNT. A total of 143 random pancreas-infiltrating B cells were utilized as donor cells for SCNT. After activation and culture of the reconstructed SCNT embryos, we then derived a single embryonic stem (ES) cell line from our B cell SCNT blastocysts, which was subsequently used to generate chimeric mice as reported previously (29, 30). A single cross of aforementioned chimeric mice with NOD-Rag1^−/−^ resulted in NOD-IgHL-Rag1^−/−^ mice, which can be directly analyzed. We refer to the SCNT BCR model presented here as B1411. Despite the i.v. injection of a CD45.1 antibody to distinguish intra-vascular from intra-pancreatic B cells, there is a possibility that the donor cell did not originate from the pancreas. Hence, we first performed histological analysis on WT NOD and B1411-Rag1^−/−^ mice to determine whether B1411 B cells could infiltrate the pancreas in the absence of T cell help at early age (6 weeks). While we found islet-infiltrating cells in all WT NOD mice (Figure 1A), we were not able to find islet-infiltrating cells in B1411-Rag1^−/−^ mice (Figure 1F). To our surprise, we were able to find peri-/intra-pancreatic lymph nodes in all B1411-Rag1^−/−^ examined (*n* = 3, Figures 1I,J representing peri-/intra-pancreatic lymph nodes), while none were observed in WT NOD mice (*n* = 3, Figure 1D,E representing mesenteric lymph nodes). As shown in Figure S1, we were also able to identify some pancreatic lobes with lymphocytic infiltrates in B1411-Rag1^−/−^ mice, while in the same pancreas other lobes appeared healthy. Compared to WT NOD (Figures 1B,C) the spleen of B1411-Rag1^−/−^ (Figures 1G,H) displayed an ill-defined, hyperplastic white pulp, especially defined through a hyperplastic marginal zone (MZ). However, the compartments looked similar at the cellular level, without a typical morphology for high B-cell turnover, which classically can be observed in germinal center reaction. While the lymph nodes of WT NOD (Figures 1D,E) showed a well-preserved normal germinal center reaction, B1411-Rag1^−/−^ mice (Figures 1I,J) showed large bright structures in every of the examined lymph nodes independent of its location (e.g., mesenteric and peri-/intra-pancreatic).

### Characterization of B Cell Receptor and Secreted Antibodies

**Figure 2:**
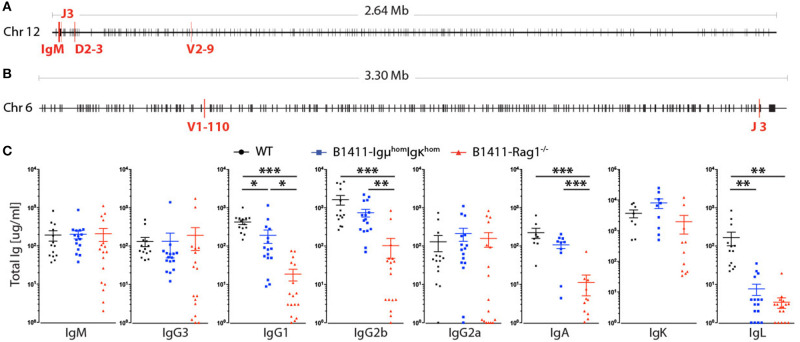
B cell receptor and antibody secretion. Identification of the V(D)J rearrangements underlying the BCR IgH- **(A)** and IgL-locus **(B)** in the SCNT-derived B1411 mouse model using useast.ensembl.org/index.html. **(C)** Scatterplots showing the antibody levels of all isotypes found in the serum of WT NOD (black circles), B1411-Igμ^hom^Igκ^hom^ (blue squares) and B1411-Rag1^−/−^ (red triangles) mice; IgG1, IgG2b and IgA were found to be consistently low in B1411-Rag1^−/−^ mice. Error bars are expressed as mean ±SEM. *indicates *p*-value < 0.05; **indicates *p*-value < 0.01; ***indicates *p*-value < 0.001.

To identify the V(D)J rearrangement underlying the B cell receptor (BCR) of B1411, we performed 5′-Rapid Amplification of C-terminal Ends (5′-RACE). We showed that B1411 expressed Igμ and Igκ, with a V2-9, D2-3, and J3 rearrangement underlying IgM (Figure 2A), and a V1-110 and J3 rearrangement underlying Igκ (Figure 2B). We also found that neither Igμ nor Igκ had undergone somatic hypermutation (SHM), meaning they were germline encoded. Given that most autoimmune BCR transgenic models have no, or low amounts of secreted antibodies, we determined immunoglobulin (Ig) levels in B1411-Rag1^−/−^ and mice homozygous for IgH and IgL of B1411, referred to as B1411-Igμ^hom^Igκ^hom^. We decided to use B1411-Igμ^hom^Igκ^hom^ mice in several experiments for two reasons. First, because B1411-Igμ^hom^Igκ^hom^ mice are homozygous for both IgH and IgL, most developing B cells should express the cognate BCR. Second, because B1411-Igμ^hom^Igκ^hom^ mice are Rag1-proficient, CD4^+^ T cells are present to provide T cell help. We analyzed the serum from WT NOD, B1411-Igμ^hom^Igκ^hom^, and B1411-Rag1^−/−^ mice for secreted antibodies. As shown in Figure 2C, we found antibodies of most isotypes in the serum of both B1411-Igμ^hom^Igκ^hom^ and B1411-Rag1^−/−^ mice. Class-switching in B1411-Rag1^−/−^ was therefore T cell independent and indicated that the cognate antigen was present in unstimulated B1411-Rag1^−/−^ mice. We found IgG1, and IgG2b and IgA to be consistently low in B1411-Rag1^−/−^ mice. We also found significantly lower levels of Igλ in B1411-Igμ^hom^Igκ^hom^ suggesting very low levels of receptor light-chain editing.

### Development of Physiological Autoimmune B Cells

**Figure 3:**
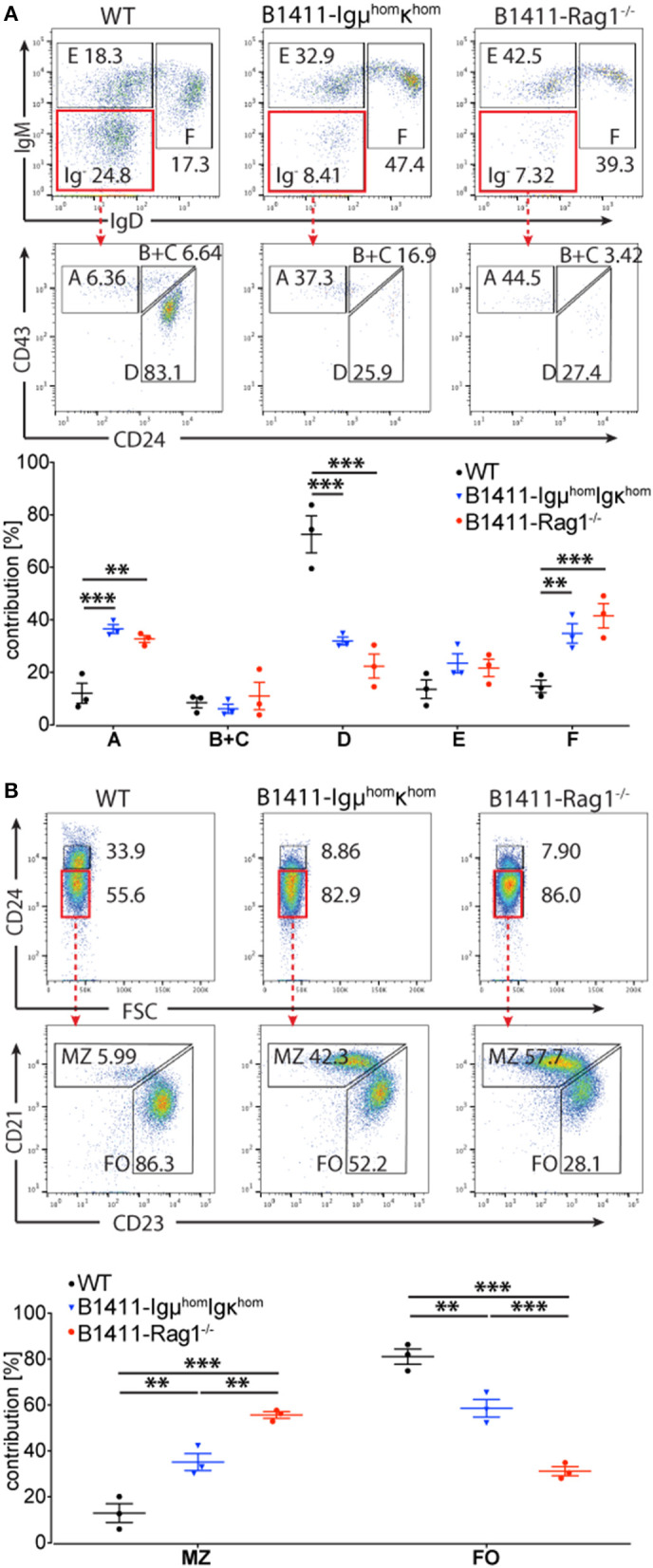
Development of autoimmune B1411 B cells. **(A)** Flow cytometric analysis of B cell development in the bone marrow showing the Hardy Fractions E and F in WT NOD, B1411-Igμ^hom^Igκ^hom^, and B1411-Rag1^−/−^ mice. The Hardy Fractions A-D were determined within the Ig^−^ population of indicated mice (top row). Dot plot showing frequencies of Hardy Fractions A-F in indicated mice (bottom row). Error bars are expressed as mean ±SEM. Initial gate on live B220^+^ CD3^−^ CD4^−^ CD8^−^ Gr-1^−^ CD11b^−^ cells. **(B)** Flow cytometric analysis of B cell development in the spleen of WT NOD, B1411-Igμ^hom^Igκ^hom^, and B1411-Rag1^−/−^ mice. Marginal Zone (MZ) and Follicular (FO) B cells were determined within the CD24^Lo^ B cell subsets of indicated mice. Scatter dot plot showing frequencies of MZ and FO in indicated mice (bottom row). Error bars are expressed as mean ±SEM. Initial gate on live B220^+^ CD3^−^ CD4^−^ CD8^−^ Gr-1^−^ CD11b^−^ cells. ***indicates *p*-value < 0.001. ** indicates *p*-value < 0.01.

Many traditional transgenic autoimmune B cell models display developmental defects (22). Given that B1411 originated from a physiological early pancreas-infiltrating B cells, we determined whether it might display certain aspects of development arrest. As shown in Figure 3A, analysis of the bone marrow from B1411-Igμ^hom^Igκ^hom^ and B1411-Rag1^−/−^ mice revealed a relative increase of Hardy Fraction F (IgM^+^ IgD^+^), and a decrease of Ig^−^ B cells compared to WT NOD mice (38). Interestingly, the IgM levels in Hardy Fraction F -as judged by mean fluorescence intensity- in B1411-Igμ^hom^Igκ^hom^ and B1411-Rag1^−/−^ were higher than those in WT NOD mice (Figure 3A). Within the Ig^−^ population, we found a relative decrease in Fraction D and a relative increase in Fraction A.

In the spleen, we found an overall decrease of immature CD24^high^ B cells in B1411-Igμ^hom^Igκ^hom^ and B1411-Rag1^−/−^ compared to WT NOD (Figure 3B). Among the transitional B cells, we found a reduced T3 fraction in B1411-Igμ^hom^Igκ^hom^ and B1411-Rag1^−/−^ (Figure S2A). Among the mature B cells, we found a relative increase in marginal zone (MZ) B cells in B1411-Igμ^hom^Igκ^hom^ (~37%) and B1411-Rag1^−/−^ (~57%) compared to WT NOD (~13%) mice, with a relative decrease in follicular (FO) B cells. Analysis of the peritoneal cavity revealed that B1411 mainly contributed to the B2 subset (Figure S2B).

### Ca-Signaling and BCR Strength in Autoimmune B Cells

**Figure 4:**
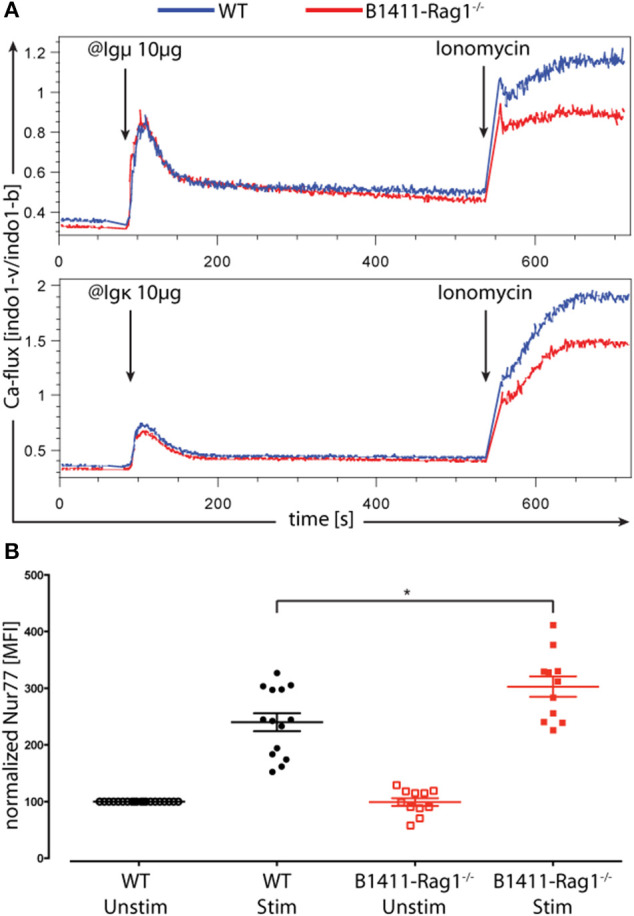
Ca-signaling and BCR strength. **(A)** Both WT NOD (stained with CFSE) and B1411-Rag1^−/−^ purified splenic B cells were mixed and stained with calcium indicator dye Indo-1. Ca-flux was determined upon addition of anti-Igμ (top) or anti-Igκ (bottom). Ionomycin was added after about 9 min. **(B)** Using endogenous Nur77-levels as indicator, the BCR strength of WT NOD (black circles) and B1411-Rag1^−/−^ (red squares) mice were determined after stimulation with anti-Igμ. Nur77 levels were normalized to unstimulated WT B-cells (black open circles); *indicates *p*-value < 0.05. Error bars are expressed as mean ± SEM.

In B cells, central tolerance is mediated through receptor editing and clonal deletion (39). It is hypothesized that many self-reactive B cells, which evade central tolerance, are silenced in the periphery through anergy. This phenomenon holds true for most traditional transgenic autoimmune BCR models (22). In order to test whether B cells from B1411 mice are anergic, we isolated splenocytes from WT NOD and B1411-Rag1^−/−^ mice and stained either one with CFSE. Subsequently, both populations were mixed and stained with calcium indicator dye Indo1. As shown in Figure 4A, upon stimulation with anti-Igμ or anti-Igκ B cells from B1411-Rag1^−/−^ responded with Ca-flux comparable to WT NOD mice.

Given that the SCNT-derived B cell mouse was generated from a pancreas-infiltrating B cell, we determined the signal strength of the underlying autoimmune BCR. BCR strength is tuned by endogenous antigen levels and is independent of inflammatory stimuli, which can be assayed directly by Nur77 expression levels (40, 41). Therefore, we measured endogenous Nur77-levels to determine the BCR strength in our autoimmune B cell model. We isolated splenic B cells from WT NOD and B1411-Rag1^−/−^ mice and activated them with anti-IgM. As shown in Figure 4B, upon stimulation B cells from B1411-Rag1^−/−^ expressed significantly higher levels of Nur77 compared to B cells from WT NOD mice indicating a higher BCR strength.

### Spontaneous Germinal Center Formation in Autoimmune B1411

**Figure 5:**
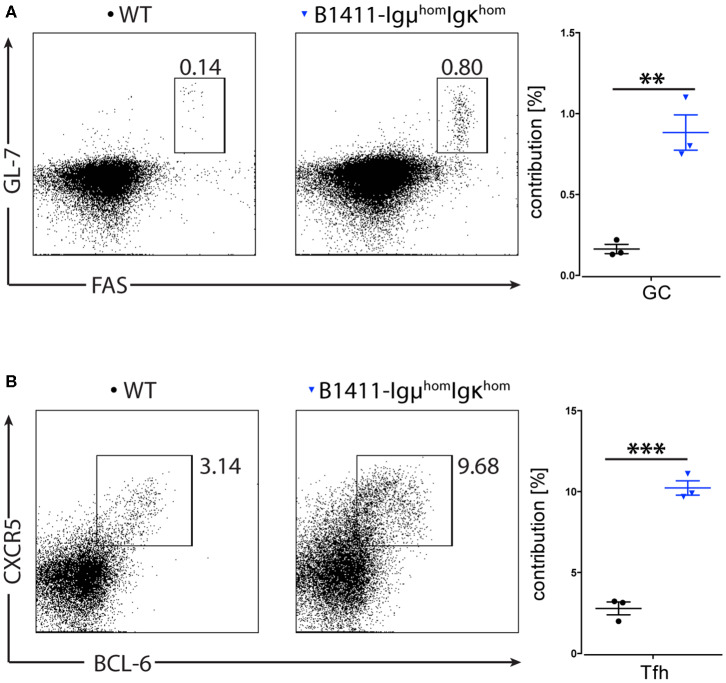
Spontaneous Germinal Center reaction. Flow cytometric analysis of germinal center (GC) B220^+^ B cells **(A)** and CD4^+^ T follicular helper (T_FH_) cells **(B)** in the spleen of WT NOD and B1411-Igμ^hom^Igκ^hom^ mice in the absence of any stimulation (left). Scatter dot plot showing frequencies of T_FH_ and GC B cells in WT NOD (black circles) and B1411-IguhomIgkhom (blue triangle). ***indicates *p*-value < 0.001. ** indicates *p*-value < 0.01. Error bars are expressed as mean ± SEM.

Given that our B1411-Rag1^−/−^ B cell model formed peri-/intra-pancreatic lymph nodes, and underwent class-switch recombination without T cell help, we reasoned that the cognate antigen should be present in untreated NOD mice and thus B1411 might spontaneously participate in germinal center (GC) reactions. To test this hypothesis, we analyzed spleen from WT NOD and B1411-Igμ^hom^Igκ^hom^ in the absence of any kind of immunization (age 8–12 weeks). As mentioned above, in B1411-Igμ^hom^Igκ^hom^ mice most B cell express the cognate BCR, and these mice possessed CD4^+^ T cells required for GC reaction. As shown in Figure 5A, we found a significant increase in GC B cells in untreated B1411-Igμ^hom^Igκ^hom^ compared to WT NOD mice. In line with our GC B cell data, we also found a significant increase in follicular helper T (T_FH_) cells in B1411-Igμ^hom^Igκ^hom^ compared to WT NOD mice (Figure 5B) indicating that the cognate autoantigen is present in untreated NOD mice.

### Transcriptional Profiling of Autoimmune B1411 Cells

**Figure 6:**
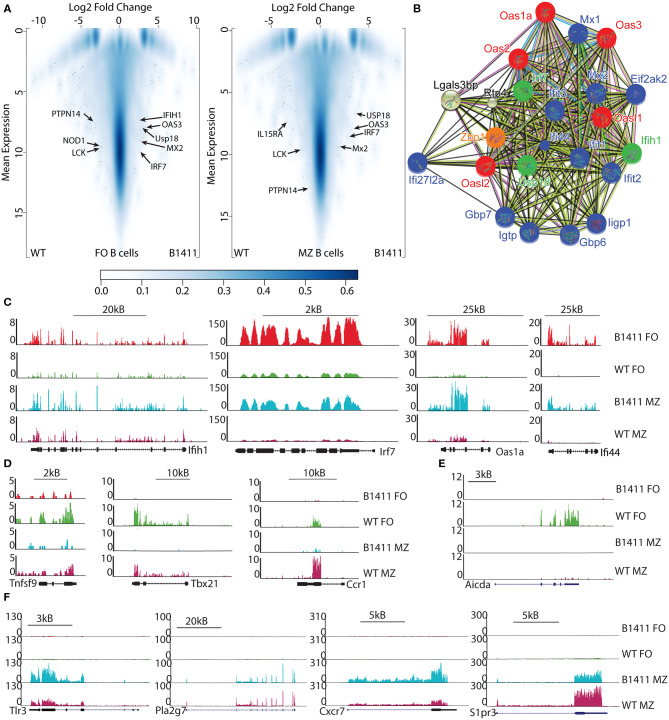
Transcriptome profiling of B1411 B cells. **(A)** Volcano plots show differentially expressing genes comparing FO (left) or MZ (right) B cells from B1411-Rag1^−/−^ and WT NOD mice. **(B)** String analysis shows differentially expressing genes enriched in protein-protein interaction network involving oligoadenylate synthase (Oas) and Interferon-stimulated gene (ISG) pathways. **(C)** UCSC browser tracks showing RNA-Seq data in FO and MZ B cells from B1411-Rag1^−/−^ and WT NOD mice highlighting selected genes that are up-regulated in B1411-Rag1^−/−^ specifically. **(D)** UCSC browser tracks showing RNA-Seq data in FO and MZ B cells from B1411-Rag1^−/−^ and WT NOD mice highlighting selected genes that are down-regulated in B1411-Rag1^−/−^. **(E)** *Aicda* expression in indicated cells. **(F)** UCSC browser tracks showing RNA-Seq data in FO and MZ B cells from B1411-Rag1^−/−^ and WT NOD mice highlighting selected genes that are up-regulated in MZ B cells.

The developmental and functional characteristics of our B1411 model were quite distinct from existing transgenic autoimmune B cell models. Particularly, the functional properties prompted us to determine whether our autoimmune B cell model executes a different transcriptional program than WT B cells. Consequently, we performed mRNA-Seq analysis to investigate the differential gene expression between FO and MZ B cells from B1411-Rag1^−/−^ mice and compared them with FO and MZ B cells from WT NOD mice. When we performed hierarchical clustering of transcriptomic data, we found that FO B cells from B1411-Rag1^−/−^ and WT mice are highly transcriptionally similar (~99% similarity), as well as between MZ B cells from B1411-Rag1^−/−^ and WT (>99% similarity). Specifically, we found 338 differentially expressing genes in FO B cells out of which 93 genes were up-regulated in B1411, and 245 genes were up-regulated in WT (Figure 6A). We also found 138 differentially expressing genes in MZ B cells, out of which 61 genes were up-regulated in B1411-Rag1^−/−^, and 77 genes were up-regulated in WT MZ B cells. In order to determine whether the differentially expressing genes are part of a protein-protein interaction network, we subjected the differentially expressing genes to the Search Tool for the Retrieval of Interacting Genes/Proteins (STRING) analysis (34). Surprisingly, in B1411-Rag1^−/−^ we found a predicted network surrounding *Irf7, Usp18*, and *Mda5* (also known as *Ifih1*) and involving various members of the oligoadenylate synthase (OAS) family and Interferon-stimulated genes (ISG) (Figures 6B,C, Figures S3, S4), while in WT FO B cells we found a predicted interaction network involving cell cycle genes (Figure S5), as well as genes involved in germinal center reactions and T cell activation, such as *Tnfsf9* (*4-1BB Ligand*), and *Tbx21* (*T-bet*) (Figure 6D).

As shown in Figure 2C, B1411-Rag1^−/−^ underwent class-switch recombination (CSR), a process involving activation-induced cytidine deaminase (AID, encoded by *Aicda*). Our histo-pathological analysis could not identify germinal center morphology in spleen and lymph nodes of B1411-Rag1^−/−^. However, we found unusual bright structures in the lymph nodes of B1411-Rag1^−/−^ involving tingible body macrophages, suggesting lymph nodes as potential location of CSR. In line with these observations, we found expression of *Aicda* only in WT FO B cells and not in FO or MZ B cells of B1411-Rag1^−/−^ (Figure 6E).

As shown in Figures 1G, 3B, we found an increased occurrence of splenic MZ B cells in B1411-Rag1^−/−^ mice both histologically and by flow cytometric analysis, respectively. This phenomenon is common to many BCR transgenic mouse models, and is more enhanced on NOD background (42). Thus, we analyzed our transcriptional data for genes that had been suggested to play an important role in executing MZ program. As shown in Figure 6F, we identified *Cxcr7, Pla2g7, S1pr3, Prf1*, and *Tlr3* as significantly overexpressed in MZ B1411-Rag1^−/−^ compared to FO B1411-Rag1^−/−^ (43–45).

### Chromatin Accessibility in Autoimmune B1411 B Cells

**Figure 7:**
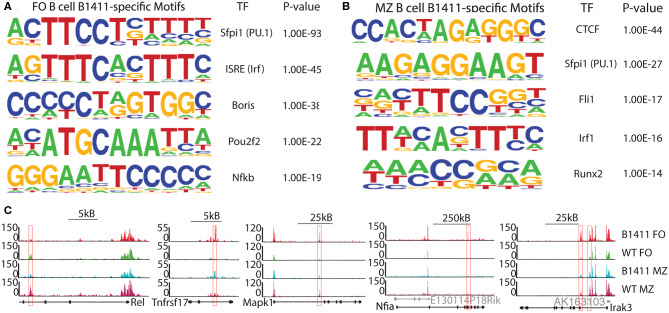
Chromatin accessibility of B1411 B cells. **(A)** Transcription factor (TF) motifs enriched in accessible chromatin sites in B1411-Rag1^−/−^ FO B cells. **(B)** TF motifs enriched in accessible chromatin sites in B1411-Rag1^−/−^ MZ B cells. **(C)** UCSC browser tracks show differential chromatin accessibility in FO and MZ B cells from B1411-Rag1^−/−^ and WT NOD mice.

Among those genes that were differentially expressing in FO B cells from B1411-Rag1^−/−^ when compared to FO B cells from WT B cells, several of them were interferon-stimulated genes and members of the OAS family. Given these novel findings we wanted to determine whether autoimmune B1411 B cells also possess a distinct epigenetic signature. Thus, to determine accessible chromatin regions, we utilized the assay of transposase accessible chromatin followed by next-generation sequencing (ATAC-Seq) (35). We found several transcription factor binding motifs that are uniquely enriched in FO B cells from B1411-Rag1^−/−^ when compared to FO B cells from WT NOD mice (Figure 7A). Among those transcription factor (TF) binding motifs were interferon-sensitive response element (ISRE) and NF-κB binding sites. In MZ B cells from B1411-Rag1^−/−^, we found a different set of transcription factor binding sites including CTCF and Irf1 binding sites (Figure 7B). As example, we showed ATAC-Seq peaks that were enriched in WT FO and MZ B cells (Rel promoter), and ones that were enriched in B cells from B1411 (Tnfrsf17, Mapk1, Nfia, and Irak3), respectively (Figure 7C).

### Glucose Tolerance Test in Autoimmune B1411 Mice

**Figure 8:**
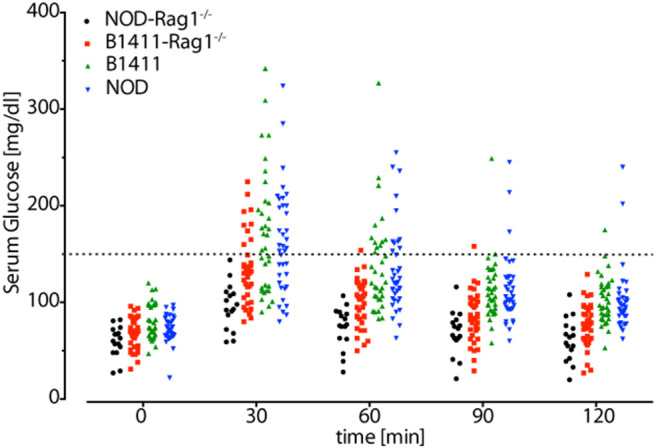
Glucose tolerance test. Fasting serum glucose levels (time = 0 min) were determined for NOD-Rag1^−/−^ (black circles), B1411-Rag1^−/−^ (red squares), B1411 (green triangles), and NOD (blue triangles) mice. Mice were injected with 2 g glucose per kg body weight and serum glucose levels were determined in 30 min intervals. Mice with serum glucose levels above 150 mg/dl (dotted line) possess impaired glucose tolerance. Each symbol represents one mouse.

It had been shown that NOD mice, which were depleted of either B cells or T cells, do not develop T1D. Thus, given that mice lacking T cells, but containing the full BCR repertoire do not develop T1D, we considered it unlikely that B1411-Rag1^−/−^ mice would develop T1D. However, we reasoned that our physiological autoimmune B1411 model might be sufficient to impair insulin secretion or function. To test this hypothesis, we performed glucose tolerance test (GTT). We used NOD-Rag1^−/−^ mice as negative controls, and WT NOD mice as positive controls. Fasting glucose levels were initially determined for NOD-Rag1^−/−^, WT NOD, B1411, and B1411-Rag1^−/−^ mice. After i.p. injection of glucose, serum glucose levels were determined in 30 min intervals. As shown in Figure 8, glucose levels of many WT NOD mice had glucose levels above 150 mg/dl. As hypothesized, many of the B1411 mice had also serum glucose levels above the threshold, indicating that B1411 in the presence of T cells can cause prediabetes. Strikingly, we also found a few B1411-Rag1^−/−^ mice with elevated serum glucose levels above 150 mg/dl, indicating that in some mice B1411 can induce prediabetes.

## Discussion

To deconvolute the involvement of B cells during the onset of T1D, we developed a novel model by using physiological early pancreas-infiltrating B cells from NOD mice as donor cells for SCNT. We hypothesized that by utilizing B cells that are physiologically involved in T1D, we could recapitulate the pathogenesis of T1D closer to reality. On a Rag1-deficient background pancreas from the resulting B1411 model showed peri-/intra-pancreatic lymph nodes that we did not observe in WT NOD mice (Figure 1).

We determined that the underlying BCR of B1411 was germline encoded IgM and Igκ with currently unknown specificity. However, previous analysis of pancreas-infiltrating B cells from prediabetic NOD mice had revealed seven Vκ gene segments that were present at frequencies of 5% or higher. While Vκ14-126 was the most dominating Vκ gene segments in the pancreas of these 9-week old prediabetic mice, Vκ1-110 was among these top seven Vκ gene segments. As mentioned above, B1411 was generated from a B cell isolated from a 6-week old pre-diabetic mouse and utilized Vκ1-110 further supporting the idea that B1411 originated from a true physiological early pancreas-infiltrating B cell. Surprisingly, in B1411-Rag1^−/−^ mice we detected antibodies of almost all isotypes in the absence of any T cell help. Histological analysis showed that WT NOD mice had well-preserved, normal germinal center reaction in their lymph nodes. Contrary, B1411-Rag1^−/−^ mice showed large bright structures in every of the examined lymph nodes (Figures 1I,J). These structures displayed a mixed cellular infiltrate, comprising lots of histiocytoid cells, lymphocytes, and tingible body macrophages. These findings strongly indicated that these bright structures might represent locations of T-cell independent immunoglobulin class switch recombination. In line with this hypothesis, macrophages had been shown to fill the gap of T-follicular helper cells, in the context of HIV mediated T-helper cell depletion (46). Thus, a potential mechanism of T-independent CSR in B1411-Rag1^−/−^ mice, could be macrophage-mediated CSR in lymph node as spleen of B1411-Rag1^−/−^ did not express any *Aicda* (Figure 6E).

Contrary to most transgenic autoimmune BCR models, B1411 did not display any signs of developmental arrest, but rather accelerated maturation, which is a feature commonly observed in non-autoimmune BCR transgenic mouse models (Figure 3) (38). In line with this, we found that B1411 displayed Ca-flux comparable to WT B cells when stimulated with anti-IgM or anti-Igκ (Figure 4). Strikingly, the Nur77-levels found in B1411 point toward a higher BCR strength compared to polyclonal B cells in WT NOD mice, which was unexpected for an autoimmune B cell. In addition, we showed that B1411-Igμ^hom^Igκ^hom^ mice were able to undergo spontaneous germinal center reaction, which is most likely due to exposure to its cognate endogenous antigen (Figure 5). Our data, demonstrating spontaneous class-switching and peri-/intra-pancreatic lymph nodes in the absence of T cell help, as well as the Ca-mobilization and spontaneous germinal center reaction argue against an anergic state in B1411 B cells. However, identification of the cognate antigen is required to formally demonstrate auto-reactivity and the lack of anergy in B1411.

Surprisingly, mRNA-Seq analysis of FO and MZ B cells from B1411-Rag1^−/−^ and WT NOD revealed an almost identical transcriptome (Figure 6). However, a closer look at differentially expressing genes in our autoimmune B1411 model revealed a unique protein-protein interaction network (Figure 6B). Within this predicted interaction of B1411, we discovered *Irf7, Usp18*, and *Ifih1* (*Ifih1* is encoding Mda5), which had been implicated to play a pivotal role in the viral etiology of T1D. Particularly, Mda5 had been linked to the onset of T1D. Although previous studies focused on the expression of *Irf7, Usp18*, and *Ifih1* in pancreatic β-cells, the presence of these disease-associated genes in our physiological autoimmune B cell model is very intriguing and suggested a so far underestimated role of these genes in B cells (47–49). In addition to *Irf7, Usp18*, and *Ifih1*, we also found various Interferon-stimulated genes (ISG) as well as members of the oligoadenylate synthase (OAS) family. Upon sensing of cytosolic dsRNA, 2′-5′-oligoadenylate synthase (OAS) proteins generate 2′-5′-linked oligoadenylates, which are second messengers leading to the activation of RNaseL (50). Among the OAS family members, Oas1 had also been linked to onset of T1D in the context of viral infections (51). Thus, our unbiased SCNT approach and resulting B1411 BCR mouse model supports current studies surrounding an important role of RNA-virus in the onset of T1D. Strikingly, our ATAC-Seq analysis demonstrated that autoimmune B cell model B1411 contained open chromatin regions enriched for ISRE and NF-κB transcription factor binding sites among others (Figure 7). Both have also been implicated to link viral infections and onset of T1D (7). Whether OAS proteins are directly involved in the onset of T1D, and the role of MDA5, USP18, IRF7 and ISGs in B cells will be determined in future experiments.

As shown in Figures 1I,J, as well as Figure 3B, B1411-Rag1^−/−^ possessed significant higher amounts of MZ B cells compared to WT NOD mice as judged by both histological examination and flow cytometric analysis. An increase in MZ B cells is often observed in BCR transgenic mice, and several candidate genes have been implicated in this bias (42). We also analyzed our RNA-Seq data in regards to genes that express significantly higher in B1411 MZ B cells compared to B1411 FO B cells. As shown in Figure 6F, we found several of previously implicated candidate genes to be enriched in MZ from B1411-Rag1^−/−^ mice, including *Cxcr7, Pla2g7, S1pr3, Prf1*, and *Tlr3*. Strikingly, *S1pr3* was among the most differentially expressed genes, and had been shown to be required in guiding B cells to the marginal zone (43–45).

It had been shown that NOD mice, which were T cell deficient but possessed a diverse BCR repertoire, do not develop overt T1D. Thus, we determined whether B1411-Rag1^−/−^ might be capable of inducing prediabetes as judged by elevated serum glucose levels during GTT. We found that few B1411-Rag1^−/−^ mice had an impaired glucose tolerance with serum glucose levels over 150 mg/dl at 30 min after i.p. injection of glucose. Of note, while the highest serum glucose levels in WT NOD and B1411-Igμ^hom^Igκ^hom^ mice surpassed 300 mg/dl, the highest levels in B1411-Rag1^−/−^ mice observed so far was 225 mg/dl. In addition, we found normal serum glucose levels (below 150 mg/dl) in almost all B1411-Rag1^−/−^ mice already at 60 min after the glucose challenge indicating mild impaired glucose tolerance rather than overt T1D. Given that B1411-Rag1^−/−^ mice possess only one BCR, the overall penetrance of impaired glucose tolerance and the variability in secreted antibodies is somewhat surprising indicating that additional factors might play a role in disease onset. Further analysis of the mechanisms leading to impaired glucose tolerance in B1411-Rag1^−/−^, the role of viral sensing, as well as identification of the cognate antigen will provide new insights into pathological mechanisms and potential treatment for T1D.

## Data Availability Statement

The datasets generated for this study can be found in the NCBI's Gene Expression Omnibus (GEO) and are accessible through GEO Series GSE114831.

## Ethics Statement

The animal study was reviewed and approved by Institutional Animal Care and Use Committee at the Scripps Research Institute.

## Author Contributions

NP, EK, MK, AN, JA, BF, CX, DN, and OK performed and analyzed experiments. MS performed histo-pathological analysis. NP, EK, MK, CX, DN, and OK wrote the manuscript.

## Conflict of Interest

The authors declare that the research was conducted in the absence of any commercial or financial relationships that could be construed as a potential conflict of interest.

## Supplementary Material

Figure S1 Histological comparison of pancreas from WT NOD and B1411-Rag1^−/−^. Representative HE-stained pancreatic section of B1411-Rag1^−/−^ mice showing lobes with lymphocytic infiltration (arrow).

Figure S2 Transitional B cells and Peritoneal Cavity. **(A)** Flow cytometric analysis of transitional B cells (T1-3) in the spleen within the immature CD24^hi^ B220^+^ population of WT NOD, B1411-Igμ^hom^Igκ^hom^ mice, and B1411-Rag1^−/−^ mice. Scatter dot plot showing frequencies of T1-3 in indicated mice. ^*^indicates *p*-value < 0.001. Error bars are expressed as mean ±SEM. **(B)** Flow cytometric analysis of B220^+^ cells in the peritoneal fluid of WT NOD, B1411-Igμ^hom^Igκ^hom^ mice, and B1411-Rag1^−/−^ mice. Scatter dot plot showing frequencies of B1a, B1b, and B2 cell in indicated mice. ^***^indicates *p*-value < 0.001. **indicates *p*-value < 0.01. Error bars are expressed as mean ± SEM.

Figure S3 Gene set enrichment analysis of FO B cells from B1411-Rag1^−/−^. Heatmap shows GSEA (Gene Set Enrichment Analysis) for genes up-regulated (<2 fold, *p*-value < 0.05) in B1411 FO compared to respective cell types in WT. GO gene sets and immunological signatures were selected to compute for overlaps between gene sets and MSigDB (Molecular Signatures Database).

Figure S4 Gene set enrichment analysis of MZ B cells from B1411-Rag1^−/−^. Heatmap shows GSEA (Gene Set Enrichment Analysis) for genes up-regulated (<2 fold, *p*-value < 0.05) in B1411 MZ compared to respective cell types in WT. GO gene sets and immunological signatures were selected to compute for overlaps between gene sets and MSigDB (Molecular Signatures Database).

Figure S5 Transcriptome analysis of WT B cells. String analysis shows differentially expressing genes enriched in protein-protein interaction network in FO B cells from WT NOD mice.
