# Supplementary figures and images for: Prediabetes Induced by a Single Autoimmune B Cell Clone
Source: Front Immunol. 2020 Jun 18;11:1073. doi: 10.3389/fimmu.2020.01073 (PMC7314986; doi:10.3389/fimmu.2020.01073)

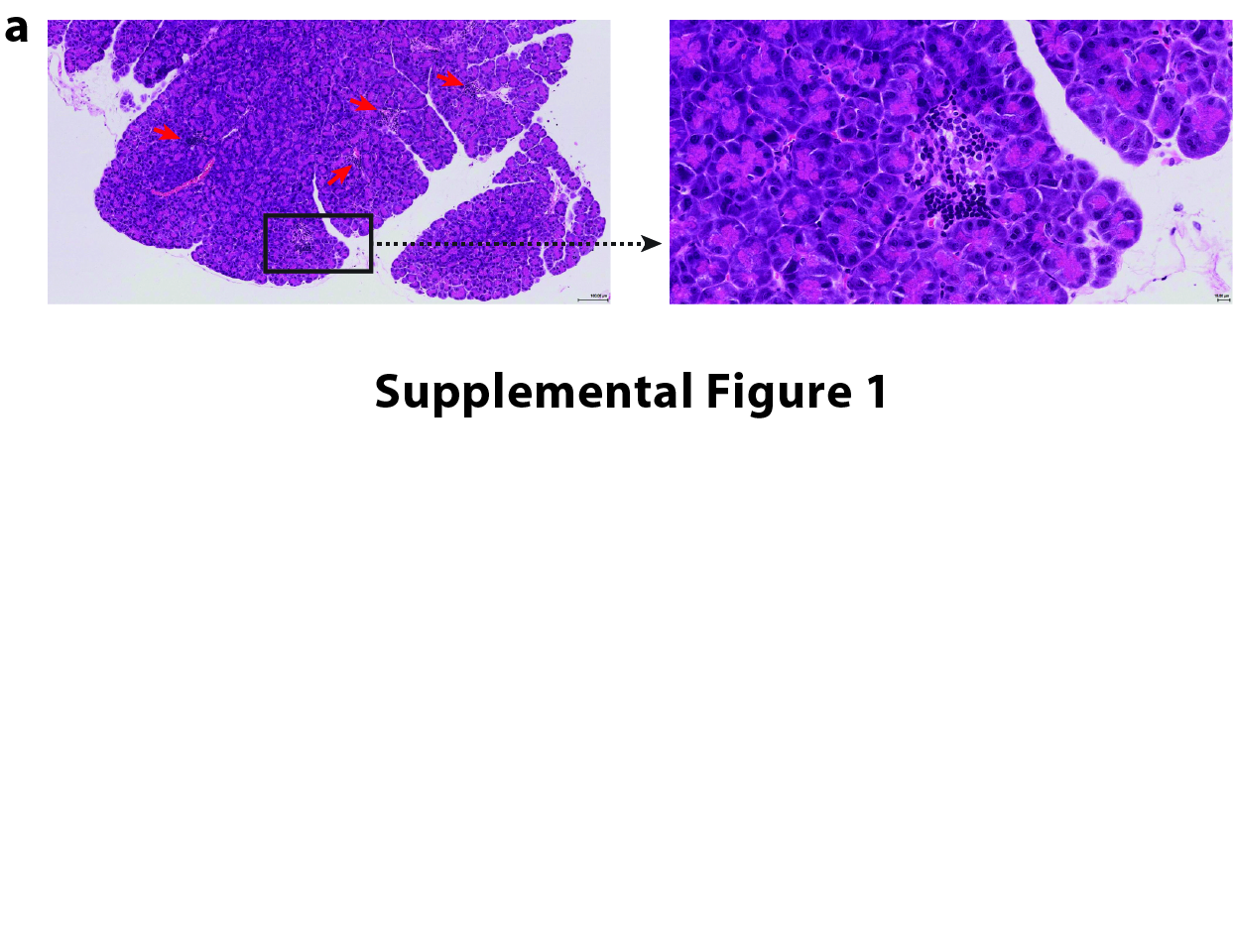

Supplement: Figure S1 — Histological comparison of pancreas from WT NOD and B1411-Rag1−/−. Representative HE-stained pancreatic section of B1411-Rag1−/− mice showing lobes with lymphocytic infiltration (arrow). [file Image_1.TIF]

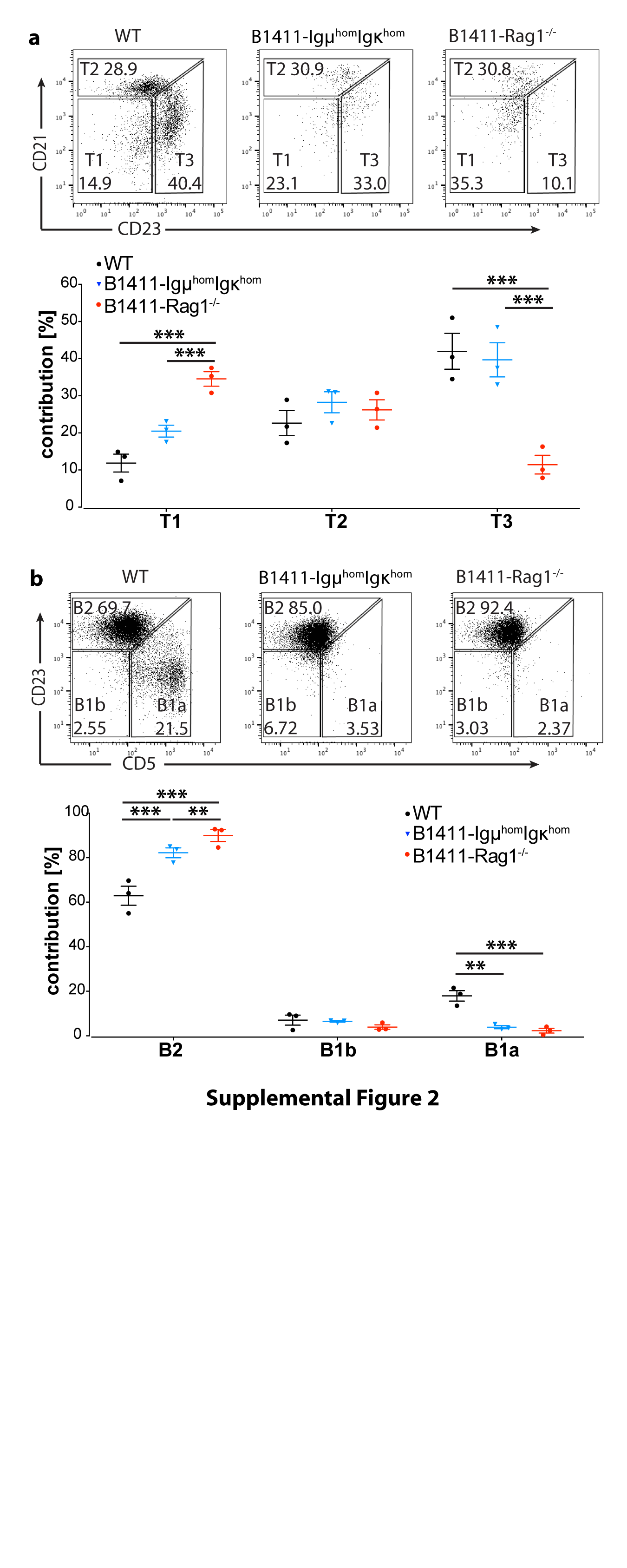

Supplement: Figure S2 — Transitional B cells and Peritoneal Cavity. (A) Flow cytometric analysis of transitional B cells (T1-3) in the spleen within the immature CD24hi B220+ population of WT NOD, B1411-IgμhomIgκhom mice, and B1411-Rag1−/− mice. Scatter dot plot showing frequencies of T1-3 in indicated mice. *indicates p-value < 0.001. Error bars are expressed as mean ±SEM. (B) Flow cytometric analysis of B220+ cells in the peritoneal fluid of WT NOD, B1411-IgμhomIgκhom mice, and B1411-Rag1−/− mice. Scatter dot plot showing frequencies of B1a, B1b, and B2 cell in indicated mice. ***indicates p-value < 0.001. **indicates p-value < 0.01. Error bars are expressed as mean ± SEM. [file Image_2.TIF]

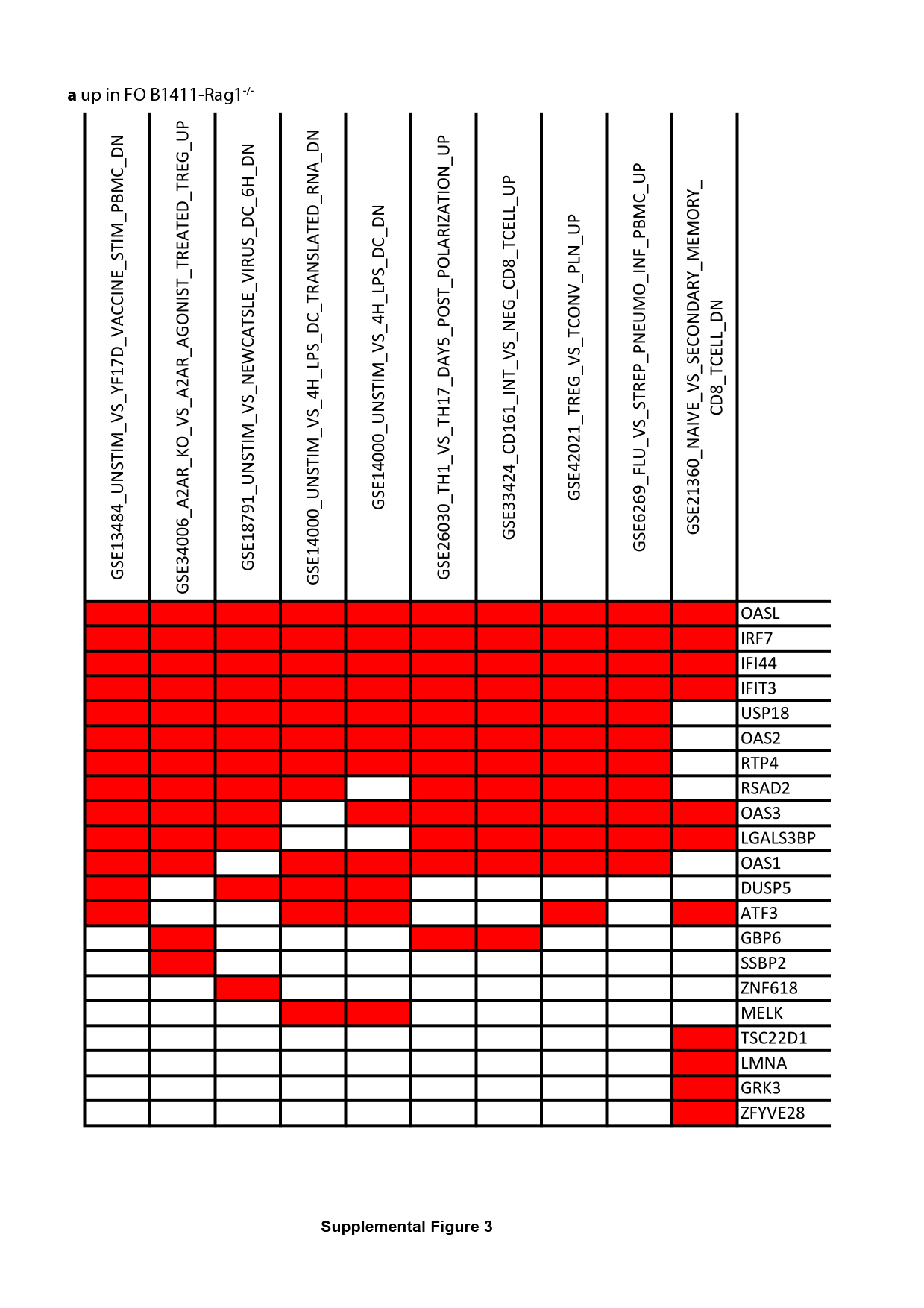

Supplement: Figure S3 — Gene set enrichment analysis of FO B cells from B1411-Rag1−/−. Heatmap shows GSEA (Gene Set Enrichment Analysis) for genes up-regulated (<2 fold, p-value < 0.05) in B1411 FO compared to respective cell types in WT. GO gene sets and immunological signatures were selected to compute for overlaps between gene sets and MSigDB (Molecular Signatures Database). [file Image_3.jpg]

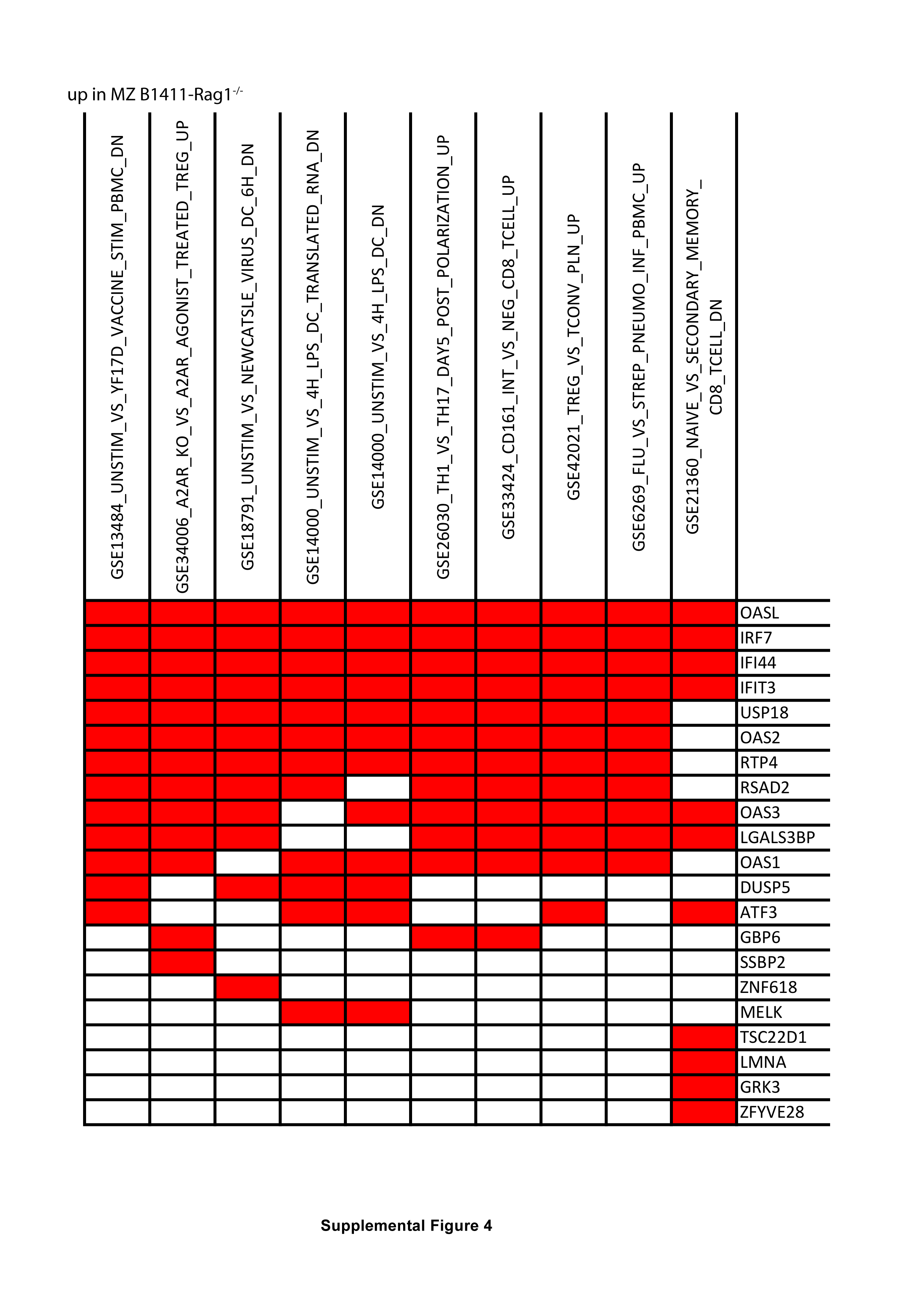

Supplement: Figure S4 — Gene set enrichment analysis of MZ B cells from B1411-Rag1−/−. Heatmap shows GSEA (Gene Set Enrichment Analysis) for genes up-regulated (<2 fold, p-value < 0.05) in B1411 MZ compared to respective cell types in WT. GO gene sets and immunological signatures were selected to compute for overlaps between gene sets and MSigDB (Molecular Signatures Database). [file Image_4.TIF]

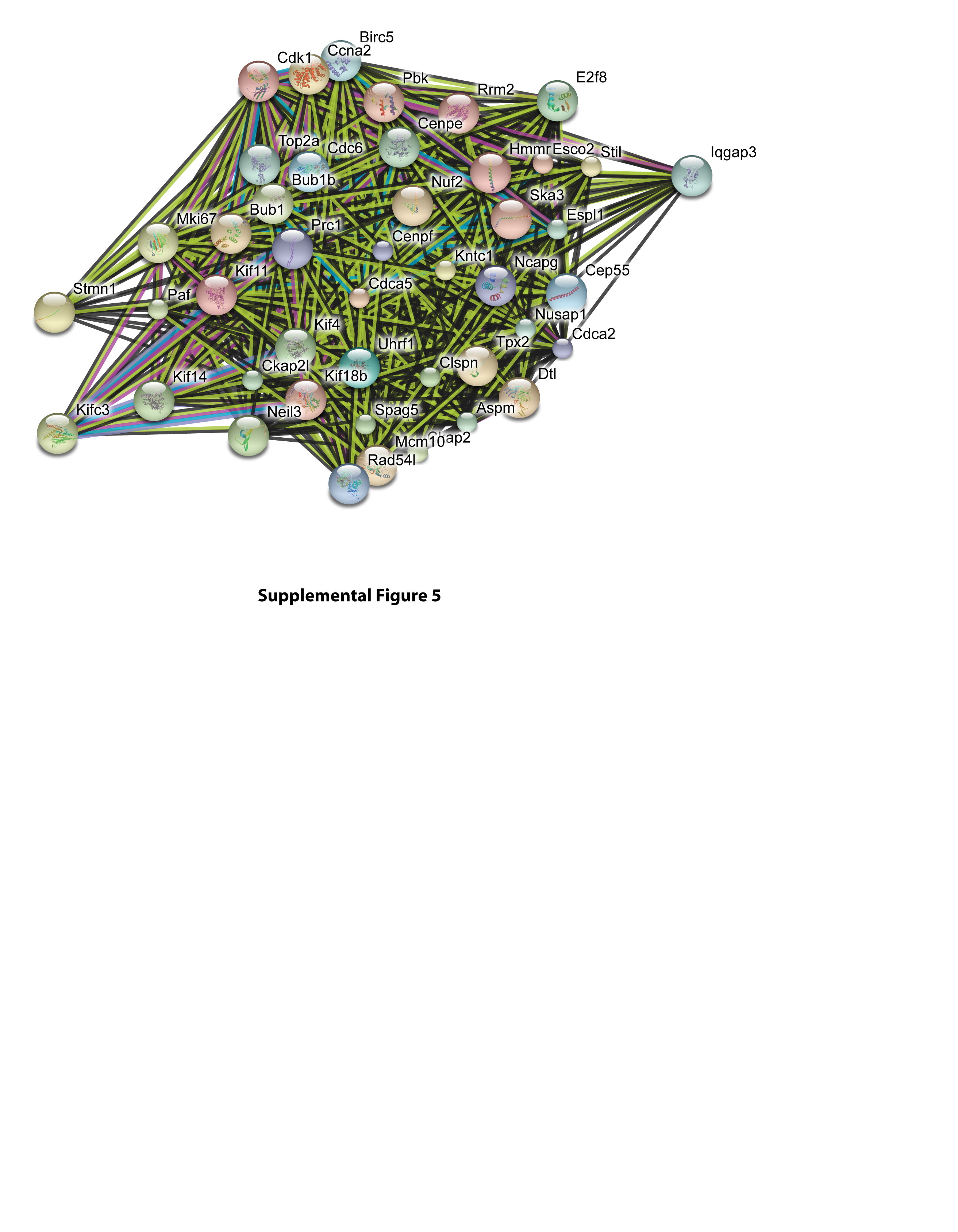

Supplement: Figure S5 — Transcriptome analysis of WT B cells. String analysis shows differentially expressing genes enriched in protein-protein interaction network in FO B cells from WT NOD mice. [file Image_5.TIF]
